# Regulation of chondrocyte gene expression by osteogenic protein-1

**DOI:** 10.1186/ar3300

**Published:** 2011-03-29

**Authors:** Susan Chubinskaya, Lori Otten, Stephan Soeder, Jeffrey A Borgia, Thomas Aigner, David C Rueger, Richard F Loeser

**Affiliations:** 1 Department of Biochemistry, Rush University Medical Center, 1653 W. Congress Parkway, Chicago, IL 60612, USA; 2 Department of Orthopedic Surgery, Rush University Medical Center, 1653 W. Congress Parkway, Chicago, IL 60612, USA; 3 Section of Rheumatology, Rush University Medical Center, 1653 W. Congress Parkway, Chicago, IL 60612, USA; 4 Klinikum Coburg GmbH, Ketschendorfer Str. 33, D - 96450 Coburg, Germany; 5 Department of Pathology, Rush University Medical Center, 1653 W. Congress Parkway, Chicago, IL 60612, USA; 6 Stryker Biotech, 35 South Street, Hopkinton, MA 01748, USA; 7 Wake Forest University School of Medicine, Medical Center Blvd, Winston-Salem, NC 27157, USA

## Abstract

**Introduction:**

The objective of this study was to investigate which genes are regulated by osteogenic protein-1 (OP-1) in human articular chondrocytes using Affimetrix gene array, in order to understand the role of OP-1 in cartilage homeostasis.

**Methods:**

Chondrocytes enzymatically isolated from 12 normal ankle cartilage samples were cultured in high-density monolayers and either transfected with OP-1 antisense oligonucleotide in the presence of lipofectin or treated with recombinant OP-1 (100 ng/ml) for 48 hours followed by RNA isolation. Gene expression profiles were analyzed by HG-U133A gene chips from Affimetrix. A cut-off was chosen at 1.5-fold difference from controls. Selected gene array results were verified by real-time PCR and by *in vitro *measures of proteoglycan synthesis and signal transduction.

**Results:**

OP-1 controls cartilage homeostasis on multiple levels including regulation of genes responsible for chondrocyte cytoskeleton (cyclin D, Talin1, and Cyclin M1), matrix production, and other anabolic pathways (transforming growth factor-beta (TGF-β)/ bone morphogenetic protein (BMP), insulin-like growth factor (IGF), vascular endothelial growth factor (VEGF), genes responsible for bone formation, and so on) as well as regulation of cytokines, neuromediators, and various catabolic pathways responsible for matrix degradation and cell death. In many of these cases, OP-1 modulated the expression of not only the ligands, but also their receptors, mediators of downstream signaling, kinases responsible for an activation of the pathways, binding proteins responsible for the inhibition of the pathways, and transcription factors that induce transcriptional responses.

**Conclusions:**

Gene array data strongly suggest a critical role of OP-1 in human cartilage homeostasis. OP-1 regulates numerous metabolic pathways that are not only limited to its well-documented anabolic function, but also to its anti-catabolic activity. An understanding of OP-1 function in cartilage will provide strong justification for the application of OP-1 protein as a therapeutic treatment for cartilage regeneration and repair.

## Introduction

Cartilage degeneration is one of the features of osteoarthritis (OA). In order to identify cellular mechanisms that drive OA progression, it is necessary to understand the interplay between anabolic and catabolic processes responsible for cartilage homeostasis under physiological and pathophysiological states. Osteogenic protein-1 (OP-1) or bone morphogenetic protein-7 (BMP-7) is one of the most potent growth factors for cartilage maintenance and repair identified thus far [1,2]. A large number of *in vivo *and *in vitro *studies have shown a high synthetic potency of human recombinant OP-1 (rhOP-1; [2]). In earlier work, we found that the inhibition of OP-1 gene expression by antisense oligonucleotides (ODNs) caused a significant decrease in aggrecan expression, aggrecan core protein synthesis, and proteoglycan (PG) synthesis, which resulted in the depletion of PGs from the cartilage matrix [3]. These findings suggest that OP-1 plays a key role in maintenance of cartilage integrity and homeostasis, but further work is needed to understand the mechanisms by which OP-1 acts at the molecular level.

In the current study, we used the Affymetrix GeneChip technology to monitor OP-1 regulation of 22,000 genes from the human genome with specific emphasis on genes that are relevant to adult articular cartilage. Those included matrix proteins, anabolic and catabolic gene products, as well as their intracellular regulators and receptors. Recently, applying the same methodology differential gene expression pattern in normal and OA cartilage tissue was identified [4]. These analyses revealed numerous interesting gene expression profiles, but *per se *did not allow elucidating cellular reaction patterns in response to defined extracellular stimuli. The goal of the current project was to evaluate the role OP-1 plays in regulating human articular cartilage homeostasis by using a gene array approach under conditions where endogenous OP-1 gene expression was inhibited by antisense ODNs ([3]; OP-1AS) or OP-1 signaling was activated and/or enhanced by rhOP-1. Key microarray findings were verified by real-time PCR and additional *in vitro *experiments of matrix synthesis and signal transduction. We found that OP-1/BMP-7 controls numerous metabolic pathways that are not limited to its direct anabolic or anti-catabolic function, but also related to cell growth, cell proliferation, differentiation, survival, apoptosis, and death.

## Materials and methods

### Materials

Dulbecco's modified Eagle's medium (DMEM), fetal bovine serum (FBS), gentamicin, Ham's F-12, lipofectin, Opti-MEM, penicillin/streptomycin/fungizone (PSF), 1X Platinum Quantitative PCR SuperMix-UDG and SuperScript III reverse transcriptase with oligo (dT)_12-18 _were purchased from Invitrogen (Carlsbad, CA, USA). Phosphorothioate ODN was custom synthesized by Oligos Etc. (Wilsonville, OR, USA). RNeasy mini kit, QIA shredder, RNase-free DNase kit and QuantiTect Primer Assay were purchased from Qiagen (Valencia, CA, USA). Real time polymerase chain reaction (PCR) primers were custom synthesized by Integrated DNA Technologies (IDT), Coralville, IA, USA. 10,000 X SYBR Green 1 was purchased from Cambrex, Rockland, ME, USA. Recombinant human rhOP-1 was kindly provided by Stryker Biotech (Hopkinton, MA, USA).

### Isolation and culture of chondrocytes

Full-thickness articular cartilage from the talus of the talocrural joint (ankle) from 12 human organ donors (age 55 to 70 years old, Collins grade 0 to 1 [5]) and from the femur of the tibiofemoral joint (knee) from two human organ donors (age 67 and 73 years old, Collins grade 2) was obtained from the Gift of Hope Organ and Tissue Donor Network (Elmhurst, IL, USA) with Institutional Review Board approval and appropriate consent within 24 hours of the donor's death. Knee cartilage was utilized for verification of the ankle cartilage results using real-time PCR. Chondrocytes were isolated by sequential digestion with pronase (2 mg/ml) for 60 minutes and collagenase P (0.25 mg/ml) overnight [6]. Chondrocytes were plated in high density monolayer culture (4 × 10^6 ^cells/well in a six-well plate) and cultured for 24 hours in 50% DMEM/50% Ham's F-12 supplemented with 10% FBS, 1% PSF, and gentamicin (50 μg/ml) for attachment prior to treatment with either antisense (OP-1 AS) or recombinant OP-1 (rhOP-1). Both treatments were administered for 48 hours in the absence of serum.

### Phosphorothioate ODNs

Antisense ODNs were designed to be complementary to sequences in the 5'- and 3'-untranslated regions of the human OP-1 messenger RNA (mRNA) sequence (XM_030621, National Center for Biotechnology Information (NCBI)) as described [3]. All verification experiments with appropriate negative controls (sense and scrambled probes) were performed in a previous study [3]. For this study, the following antisense ODN was used: 5'-GGC-GAA-CGA-AAA-GGC-GAG-TGA-3' (position 237-257).

### Treatment groups

Chondrocyte cultures were divided into three experimental groups and treated for 48 hours as follows: 1) transfected with OP-1 AS in the presence of 10 μg/ml lipofectin [3]; 2) treated with 100 ng/ml of rhOP-1; and 3) culture control (no treatment, no serum).

### RNA Isolation

Total cellular RNA was isolated using the RNeasy Mini Kit, following lysis of the cells with a Qia shredder [7] and included an on-column DNase digestion, according to the manufacturer's instructions (Qiagen). All samples were stored at -80°C until analyzed.

### Microarray and pathway analysis

Gene expression profiles were analyzed by HG-U133A gene chips from Affimetrix (accession number: E-MTAB-571). At least 10 μg of RNA/per experimental group was required for analysis. Therefore, the RNA was pooled from donors in order to have sufficient RNA and to reduce donor-to-donor variations. Cells from all 12 donors were treated with each experimental condition. The microarray data collection was in compliance with the Minimum Information About Microarray Experiments standard [8]. The quality of the RNA was checked by the Agilent Bioanalyzer (Agilent Technologies, Inc., Santa Clara, CA, USA), and the quality of the hybridization image was checked by the affyPLM model [9]. To deal with the technical variation, each gene was measured by 11 different probes on the Affymetrix U133A microarray. A statistical model at the probe-level was used to identify the differentially expressed genes. To estimate the variance more efficiently with a small sample size, we utilized an empirical Bayesian correction of the linear model [10]. Statistical significance was considered with a *P-*value of *P *< 0.001 and fold change larger than 1.5-fold between the treatment group and corresponding control. All the data analysis was conducted using the Bioconductor/R package [11]. To interpret the biological significance of differentially expressed genes, a gene ontology analysis was conducted using DAVID/EASE [12].

### Pathway analysis and classification by gene ontology

Regulated genes (R > 1.5-fold, *P *< 0.001) were used as input for both analyses. The ingenuity pathway analysis system [13] was used to project genes onto known biological pathways (canonical pathways). The system determines a significance value for each pathway based on an F-statistics that the input-genes occur randomly within this pathway. Grouping of genes was done by computing over-representation of regulated genes in gene ontology (GO) classes [14]. Statistical analysis consisted of 1) analysis of differentially expressed genes under a single experimental condition in comparison to the corresponding control (up- or down-regulated in the presence of OP-1 antisense or rhOP-1); 2) analysis of differentially expressed genes when comparison is made between two treatments (OP-1 antisense and rhOP-1); and 3) gene ontology, when changes were analyzed within a family of genes according to their function (comparison was made between single treatment and control or between both treatments). Selected gene array results were verified experimentally *in vitro *or by real-time PCR.

### Validation experiments -quantitative real time PCR

Selected gene array results were verified by real-time PCR. SuperScript III reverse transcriptase with oligo (dT)_12-18 _was used to transcribe 4 μg of isolated total RNA into complementary DNA (cDNA) in a total volume of 20 μl according to the manufacturer's instructions (Invitrogen). Real time PCR primer sets specific for human β-actin, *GAPDH, gremlin-1, IL-6, IL-8, and LIF-1 *(Table 1) were designed using the PrimerQuest program (Integrated DNA Technologies, Inc., Coralville, Iowa, USA). The specificity of the primers was verified by testing in BLAST searches [15]. Real time PCR primer sets specific for human *18SrRNA *and *BMP-2 *were purchased from Qiagen. Real time PCR was performed using the Smart Cycler System (Cepheid, Sunnyvale, CA, USA). Each 50 μl reaction mixture contained 1X Platinum Quantitative PCR SuperMix-UDG, 0.5X Smart Cycler additive reagent (0.1 mM Tris, pH 8.0; 0.1 mg of bovine serum albumin per ml, 75 mM trehalose, and 0.1% Tween 20), 0.5X SYBR Green 1 (vendor stock 10,000X; Cambrex, Rockland, ME), 0.2 μM each of forward and reverse primer (IDT primers) or 1X QuantiTect primers (Qiagen primers) and 1 μl cDNA (1*8SrRNA*, β-actin, *BMP-2, GAPDH, gremlin-1, IL-6, IL-8*) or 2 μl cDNA (*LIF-1*). Cycling parameters were: preheat at 60°C for 120 seconds then 95°C for 120 seconds followed by 40 three-step cycles of 95°C for 15 seconds, various annealing temperatures and times (Table 1) and 72°C for 30 seconds. After the last amplification cycle, PCR products were analyzed by melting curve analysis in the Smart Cycler by slowly increasing the temperature to 95°C. The reactions were run in triplicate with appropriate controls (no cDNA template). The data were analyzed by using the Cepheid Smart Cycler software (version 2.0c) and reported as threshold cycle (C_t_). Change in gene expression was calculated as fold change = 2^-Δ(ΔCt)^, where Δ(ΔC_t_) = (C_t _sample - C_t _housekeeping gene) - (C_t _control - C_t _housekeeping gene).

**Table 1:** Sequence of primers for quantitative real time PCR

Primer	Orientation	Sequence	Annealing temp and time	Accession no.
*18SrRNA*		Qiagen QuantiTect Primer Assay	62°C, 40 sec	[GenBank:X03205]
*β-actin*	Forward	5'-TCCATCATGAAGTGTGACGTGGAC-3'	62°C, 40 sec	[GenBank:NM_001101]
	Reverse	5'-TTGATCTTCATTGTGCTGGGTGCC-3'		
*BMP-2*		Qiagen QuantiTect Primer Assay	60°C, 40 sec	[GenBank::NM_001200]
*GAPDH*	Forward	5'-TGGACTCCACGACGTACTCAG-3'	62°C, 40 sec	[GenBank:NM_002046]
	Reverse	5'-CGGGAAGCTTGTCATCAATGGAA-3'		
*Gremlin-1*	Forward	5'-ATACCTGAAGCGAGACTGGTGCAA-3'	64°C, 40 sec	[GenBank:NM_013372]
	Reverse	5'-AACAGAAGCGGTTGATGATGGTGC-3'		
*IL-6*	Forward	5'-GTCAATTCGTTCTGAAGAGGTGAGT-3'	64°C, 40 sec	[GenBank:NM_000600]
	Reverse	5'-CCCCAGGAGAAGATTCCAAAGATG-3'		
*IL-8*	Forward	5'-AGACATACTCCAAACCTTTCCACCC-3'	58°C, 30 sec	[GenBank:NM_000584]
	Reverse	5'-ATTTCTGTGTTGGCGCAGTGTGGT-3'		
*LIF-1*	Forward	5'-TAAGGAGGCCTCGCAGGATGTC-3'	64°C, 30 sec	[GenBank:NM_002309]
	Reverse	5'-TAGTCGTGTACCTTGGCACCTC-3'		

### Statistical analysis for real-time

PCR Data are expressed as mean +/- standard deviation. Statistical significance was assessed by the Student *t-*test and *P-*values < 0.05 were considered significant.

## Results

### Microarray analysis: overview of data

GeneChip (HG-U133A) expression data from un-stimulated, rhOP-1 and OP-1AS treated chondrocytes maintained in high-density monolayer culture were generated. For the analysis of the expression data we used a three step analytical strategy: (I) processing of raw intensity values and normalization of profiles, (II) examination of expression levels of gene categories that are relevant to articular cartilage, and (III) comparison of gene expression changes between the two treatments - OP-1AS to knockdown endogenous OP-1 expression vs. addition of exogenous rhOP-1.

**Figure 1:**
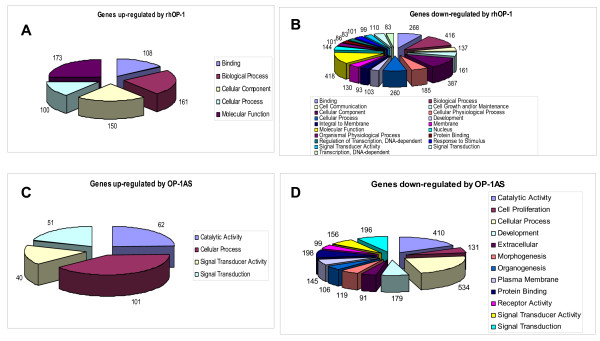
**Schematic representation of genes grouped according to their function**. **A**, genes up-regulated by treatment with recombinant OP-1; **B**, genes down-regulated by treatment with recombinant OP-1; **C**, genes up-regulated by OP-1 antisense treatment; **D**, genes down-regulated by OP-1 antisense treatment.

Analyzing the number of differentially expressed genes (fold changes of larger than 1.5 and corresponding *P*-values < 0.001 compared to control) after rhOP-1 or OP-1AS, we found that rhOP-1 modulated expression of 4,057 genes, while OP-1AS treatment modulated expression of only 2,618 genes respectively. More genes were down-regulated than up-regulated by either treatment: rhOP-1 down-regulated 3,365 genes vs 692 genes that were up-regulated; while OP-1AS down-regulated 2,364 genes and up-regulated only 254 genes. The functional groups of genes modulated by lack or excess of OP-1 are depicted in Figure 1. RhOP-1 primarily controlled genes responsible for molecular function, biological processes, and cellular components, while OP-1AS primarily affected genes controlling cellular processes and catalytic activity. Interestingly, either treatment up-regulated fewer functional groups than the number that were down-regulated (Figure 1). For example, rhOP-1 induced only five functional groups vs four induced by OP-1AS; while rhOP-1 down-regulated 19 functional groups vs 12 down-regulated by OP-1AS. When the results were compared between the two treatments, we found that very few gene groups with the same function were differentially regulated by both treatments (Figure 1). Groups regulated by both OP-1 conditions included the genes responsible for cellular processes (the same number of genes were up-regulated by either treatment, 100 vs 101), development, protein binding, signal transducer activity and signal transduction.

### Analysis of catabolic genes: cytokines and their regulators

**Figure 2:**
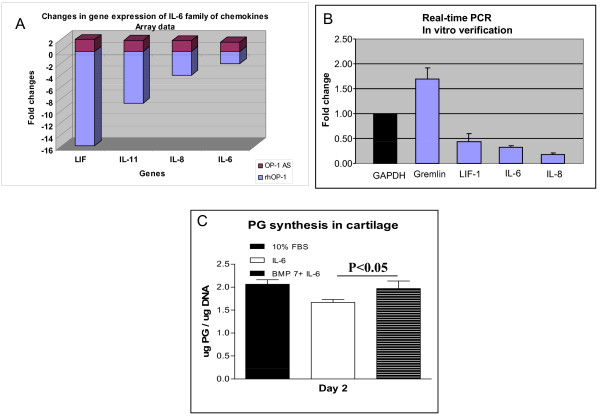
**Association between OP-1 and IL-6 family of chemokines**. **A**, Effect of lack (OP-1 antisense oligo) or excess of OP-1 (treatment with recombinant protein, 100 ng/ml, 48 hours) on gene expression of IL-6, IL-8, IL-11, and LIF in chondrocytes cultured in monolayers. Graphical representation of gene array data. **B**, Real time PCR of in vitro verification experiments, where knee chondrocytes cultured in monolayers were treated for 48 hours with the same dose of recombinant OP-1. The graph illustrates an inhibition of LIF, IL-6, and IL-8 gene expression. **C**, verification experiments with metabolic study. Proteoglycan synthesis measured in chondrocytes cultured in alginate beads and treated for 48 hours with 100 ng/ml IL-6 (in the presence of 150 ng/ml soluble IL-6 receptor) or the combination of IL-6 and OP-1 (100 ng/ml). Data were normalized to the DNA content and compared to 10% FBS control. OP-1 was able to overcome an inhibitory effect of IL-6 on PG synthesis.

Previously, we showed that OP-1 was able to counteract the catabolic activity of IL-1β [16,17] and other catabolic mediators such as fragments of cartilage matrix, fibronectin and hyaluronan [17-20]. Therefore, it was of interest to determine the effects of OP-1 on genes regulating pro-catabolic activity. Consistent with an anti-catabolic function for OP-1, a broad spectrum of genes with various pro-catabolic activities (cytokines and their regulators, matrix degrading proteinases, apoptosis-related genes, neuromediators, transcription factors, and so on) were modulated by OP-1. Multiple cytokines and chemokines, in particular members of the IL-6 family, (Figure 2), as well as their receptors and regulators of their activity (Tables 2 and 3) were found to be regulated by OP-1. Interestingly, among these mediators only members of the IL-6 family (leukemia inhibitory factor (*LIF), IL-11, IL-8*, and *IL-6*) were differentially regulated by the two treatment conditions: rhOP-1 down-regulated *LIF *expression by more than 15-fold, *IL-11 *expression by more than eight-fold, *IL-8 *gene by four-fold and *IL-6 *by two-fold, respectively (Figure 2A). Likewise, when endogenous OP-1 was inhibited by OP-1AS, expression of these four chemokines was elevated by about two-fold indicating a tight association between OP-1 levels and expression of members of the IL-6 family. Verification experiments of gene array findings included both real-time PCR analysis and *in vitro *metabolic tests (Figure 2). These tests confirmed that when chondrocytes in high-density monolayer cultures were treated with rhOP-1 for 48 hours, gene expression of *LIF, IL-6*, and *IL-8 *was inhibited as detected by real-time PCR, although the magnitude of changes was different from those identified by gene array (Figure 2A, B). In metabolic studies, we also found that OP-1 could overcome an inhibitory effect of IL-6 on PG synthesis in chondrocytes cultured in alginate beads (Figure 2C). In addition, our previous studies showed an ability of OP-1 to inhibit mRNA expression of *IL-1, IL-6, IL-8*, and other cytokines in primary and immortalized chondrocytes [17].

**Table 2:** Changes in chemokines, cytokines, and their receptors

Gene	rhOP-1 vs Cntr	OP-1AS vs Cntr	
	fold change	fold change	**Accession no**.
*LIF*	15.86↓	2.26↑	[GenBank:NM_002309]
*IL-11*	8.69↓	1.82↑	[GenBank:NM_000641]
*IL-8*	4.01↓	1.80↑	[GenBank:NM_000584]
*IL-6*	2.09↓	1.60↑	[GenBank:NM_000600]
*IL-5Rα*	2.47↑	2.40↑	[GenBank:NM_000564]
*TNF-α induced protein 6*		2.14↓	[GenBank:NM_007115]
*TNF-α induced protein-3*	2.02↓	1.60↑	[GenBank:NM_006290]
*TNF-R12*	1.79↓		[GenBank:NM_016639]
*TNF-R9*	1.73↓	1.57↓	[GenBank:NM_001561]
*TNF-R5*	1.88↑		[GenBank:NM_001250]
*TNF-13*		1.69↓	[GenBank:NM_003808]
*IL1-R1*		1.59↓	[GenBank:NM_000877]
*TNF-R11B (osteoprotegerin)*		1.58↓	[GenBank:NM_002546]
*IL-13Rα1*		1.55↓	[GenBank:NM_001560]
*IL-12β*	1.74↓		[GenBank:NM_002187]
*IL-1R accessory protein-like 1*	1.64↓		[GenBank:NM_014271]
*TNF-R6*		2.08↓	[GenBank:NM_000043]

**Table 3:** Changes in the mediators of IL-6 signaling pathway

	rhOP-1 vs Cntr	OP-1AS vs Cntr	
	fold change	fold change	**Accession no**.
**Genes from IL-6 signaling pathway**			
*ELK-1*	1.89↓		[GenBank:NM_005229]
*IL-6*	2.09↓	1.60↑	[GenBank:NM_000600]
*IL-6R*	1.81↓		[GenBank:NM_000565]
*IL-6 signal transducer *(oncostatin M receptor)		1.63↓	[GenBank:NM_002184]
*STAT1*	2.42↓		[GenBank:NM_007315]
*NFκBIα*	1.86↓	1.58↑	[GenBank:NM_020529]
*Protein inhibitor of activated STAT3*		1.84↓	[GenBank:NM_006099]
*STAT6*		1.53↓	[GenBank:NM_003153]
*MAP 3 kinase 7*		1.67↓	[GenBank:NM_003188]
*MAPK 14*		1.52↓	[GenBank:L35253]
*MAPK1*	1.55↓		

In analyzing the relationship between treatments to modulate OP-1 and the expression of genes in the IL-6 signaling pathway, we found that OP-1 not only regulates expression of the IL-6 family of cytokines but also controls expression of their receptors and downstream intracellular mediators including signal transducers and activators of transcription (STATs), mitogen activated protein (MAP) kinases, and transcription factors. This suggests OP-1 inhibits IL-6 signaling at multiple levels (Table 3). Among other genes that either regulate cytokine activity or mediate their signaling, the most affected by OP-1 were the receptors for IL-1β and tumor necrosis factor alpha (TNF-α) (see Table 2) as well as TNF-α inducible protein. Although under the experimental conditions expression of TNF-α and IL-1β genes was not influenced by OP-1, previous studies showed that injection of OP-1 into nucleus pulposus inhibited production of autocrine TNF-α and IL-1β elevated in response to injurious compression of the intervertebral discs [21] proving an association between OP-1 and signaling pathways of the above mentioned cytokines. In addition, several other studies have provided evidence of an ability of OP-1 to regulate either IL-1β induced responses or IL-1β downstream signaling [16-18,22,23].

### Analysis of catabolic genes. Neuromediators

Previous studies have provided evidence that OP-1 may regulate mediators of pain-related behavior and their activation in response to injurious compression of the intervertebral disc and acute cartilage trauma [24-26]. We also reported that injection of OP-1 into nucleus pulposus down-regulated substance P expression [26], bradykinin and bradykinin inducible receptor β1 [26]. Therefore, it was of interest to examine expression of neuromediators and their receptors in the present array study. After stimulation for 48 hours with rhOP-1, expression of the receptors of bradykinin and substance P was down-regulated (Table 4). Both receptors of bradykinin, constitutively expressed β2 and inducible β1, were down-regulated by the treatment with OP-1. Expression of the β1 receptor was differentially regulated under conditions of excess and lack of OP-1, that is, treatment with rhOP-1 inhibited gene expression of this receptor by 1.85-fold, while its expression was up-regulated by 1.59-fold when endogenous OP-1 expression was inhibited by antisense oligonucleotides. These results are consistent with previous data on the protein level in an *in vivo *model of disc herniation, where injection of OP-1 into the nucleus pulposus completely abolished bradykinin receptor β1 [26]. Although by gene array we did not identify significant changes in the expression of bradykinin and substance P at the time point tested here, we found changes in substance P receptor and its precursor. We also found that OP-1 inhibited expression of nerve growth factor-β by almost two-fold.

**Table 4:** Changes in neuromediators and their receptors

	rhOP-1 vs Cntr	OP-1AS vs Cntr	
	fold change	fold change	**Accession no**.
Bradykinin Rβ1	1.85↓	1.59↑	[GenBank:NM_000710]
Bradykinin Rβ2	1.68↓		[GenBank:NM_000623]
Tachykinin R1	1.64↓		[GenBank:NM_001058]
Nerve growth factor-β	1.93↓		[GenBank:NM_002506]
Tachykinin1 precursor (Substance K, Substance P)	2.26↓		

### Analysis of catabolic genes: Transcription factors

Besides cytokines and their receptors, OP-1 also affected gene expression of transcription factors that regulate cytokine signaling. Previously, in normal primary and immortalized chondrocytes, we found that OP-1 inhibits activation of the nuclear factor kappa-light-chain-enhancer of activated B cells (NF-κB) and activator protein-1 (AP-1) transcription factors [17]. Here, expression of a large set of transcription factors was found to be modulated by OP-1 (Table 5). In addition to common factors such as *NF-κB, STAT1 *and *STAT6*, gene array also discovered factors that repress IL-2 expression, p38 interacting protein, *Runx1*, and others. The majority of these transcription factors regulate directly or indirectly (as p38 interacting protein) transcriptional responses induced by various pro-inflammatory mediators (IL-1β, IL-6, matrix fragments). Others, like *Runx1*, are involved in the process of chondrogenesis. To further demonstrate the effect of OP-1 on activation of transcription factors, we treated cultured cells and found that OP-1 was able to at least partially inhibit activation of NF-κB in primary chondrocytes pre-treated with IL-1β or activation of Stat-1 in chondrocytes treated with IL-6 and IL-6 soluble receptor (data not shown).

**Table 5:** Changes in transcription factors

	rhOP-1 vs Cntr	rhOP-1AS vs Cntr	
	fold change	fold change	**Accession no**.
Transcription factor 8 (represses IL-2 expression)	3.28↓	2.97↓	[GenBank:NM_030751]
*NF-κB2*	2.77↓		[GenBank:NM_002502]
*STAT1*	2.42↓		[GenBank:NM_007315]
Transcription factor AP-2α	2.07↓	1.52↓	[GenBank:NM_003220]
Suppression of tumorigenicity	2.04↓	2.20↓	[GenBank:NM_013437]
*Runx1*	1.89↓	1.64↓	[GenBank:NM_001754]
*NFκBIα*	1.86↓	1.58↑	[GenBank:NM_020529]
*NFYβ*	1.68↓	1.75↓	[GenBank:NM_006166]
Activating transcription factor 7	1.66↓		[GenBank:NM_006856]
MADS box transcription enhancer factor 2-d	1.65↓		[GenBank:NM_005920]
Upstream transcription factor 2, c-fos interacting	1.58↓		[GenBank:NM_003367]
Transcription factor (p38 interacting protein)	1.57↓		[GenBank:NM_017569]
MADS box transcription enhancer factor 2-C	1.60↑	1.94↓	[GenBank:NM_002397]
Protein inhibitor of activated *STAT3*		1.84↓	[GenBank:NM_006099]
Ubiquitin-like 1 (sentrin)		1.60↓	[GenBank:NM_003352]
*STAT6*		1.53↓	[GenBank:NM_003153]

### Analysis of catabolic genes: Matrix degrading proteases, cathepsins, and apoptosis-related genes

Among other catabolic genes influenced by OP-1 were the matrix metalloproteinases (MMPs), cathepsins, and a number of proteases with various modes of action (Table 6). Thus, expression of membrane type-1 MMP (*MMP-14*) was inhibited by rhOP-1 by 1.6-fold (*P *< 0.001) along with tissue inhibitor of metalloproteinases (*TIMP)-3 *(2.06-fold, *P *< 0.001). At the same time, expression of *MMP-2 *(gelatinase A), which is activated by MMP-14 [24], was not affected by rhOP-1, but was down-regulated by OP-1AS (2.31-fold, (*P *< 0.001) as well as was *MMP-9 *(gelatinase B) (1.5-fold). Interestingly, the same positive correlation was found between the levels of OP-1 and expression of another TIMP, *TIMP-4*, which was decreased by 1.7-fold in the OP-1AS group confirming its association with MMP-2 [25]. Parallel changes were observed in other types of proteases, such as a disintegrin and metalloproteinases (ADAM)-9, 10, and 28. Their gene expression was down-regulated under OP-1AS from 2.34 to 1.75-fold. Treatment of chondrocytes with rhOP-1 inhibited expression of ADAM-15,-19, as well as urokinase type plasminogen activator, its receptor, and transglutamianse-2. There were also some proteinases that were up-regulated by rhOP-1: *ADAM-TS7, ADAM-TS12*, and tissue specific plasminogen activator suggesting that perhaps these proteins are involved in anabolic/remodeling processes.

**Table 6:** Changes in proteases and their inhibitors

	rhOP-1 vs Cntr	rhOP-1AS vs Cntr	
	fold change	fold change	**Accession no**.
*Bcl-2*	2.45↓		[GenBank:NM_001191]
Caspase 4, apoptosis-related cysteine protease	2.11↓	1.59↓	[GenBank:NM_001225]
Programmed cell death 8 (apoptosis-inducing factor)	1.70↓		[GenBank:NM_004208]
Calpain 9	1.55↓		[GenBank:NM_006615]
Caspase 6		2.18↓	[GenBank:NM_001226]
Caspase 8		1.82↓	[GenBank:NM_001228]
Caspase 2		1.50↑	[GenBank:NM_001224]
***MMPs and inhibitors***			
*TIMP-3*	2.06↓		[GenBank:NM_000362]
*MMP-14*	1.55↓		[GenBank:NM_004995]
*MMP-2*		2.31↓	[GenBank:NM_004530]
*TIMP-4*		1.69↓	[GenBank:NM_003256]
*MMP-9*		1.50↓	[GenBank:NM_004994]
***ADAM and ADAMTS***			
*ADAM-19*	1.83↓		[GenBank:NM_023038]
*ADAM-15*	1.51↓		[GenBank:NM_003815]
*ADAMTS-12*	1.88↑	2.03↑	[GenBank:NM_030955]
*ADAMTS-7*	1.58↑		[GenBank:NM_014272]
*ADAM-10*		2.34↓	[GenBank:NM_001110]
*ADAM-28*		1.63↓	[GenBank:NM_014265]
*ADAM-9*		1.61↓	[GenBank:NM_003816]
*ADAM-7*		1.56↑	[GenBank:NM_003817]
***Cathepsins***			
Cathepsin B		2.14↓	[GenBank:NM_001908]
Cathepsin C		1.75↓	[GenBank:NM_001814]
Cathepsin S		1.75↓	[GenBank:NM_004079]
***Other proteases***			
Transglutaminase 2	2.10↓		[GenBank:NM_004613]
Plasminogen activator-urokinase	1.57↓		[GenBank:NM_002658]
Tissue Plasminogen Activator	1.56↑		[GenBank:NM_000930]

Among the proteases that were also regulated by OP-1 were cathepsins B, C, and S. So far, these lysosomal cysteine proteases have been less studied in cartilage, though cathepsin C appears to be a central coordinator for activation of many serine proteases in immune/inflammatory cells [29], while cathepsin B was thought to play an important role in the development of osteoarthritis [30]. Expression of all three cathepsin genes was down-regulated under OP-1AS.

A previous study on acute impact injury *in vivo *[31] strongly suggested an anti-apoptotic effect of OP-1 in post-traumatic OA. Therefore, we expected that OP-1 may control genes involved in apoptosis-related processes. We found that rhOP-1 inhibited program cell death 8 gene (apoptosis-induced factor), *Bcl-2 *gene and the calpain-9 gene (Table 6). However, the key caspases that trigger and promote cell death by apoptosis were not affected. During the absence of OP-1 (antisense treatment), expression of caspases 8, 9, and 6 were inhibited and only caspase 2 was elevated (Table 6). The reason for a down-regulation of the apoptosis-related genes under conditions where OP-1 is lacking is not clear, but may be a response to help avoid cell death.

### Analysis of anabolic genes: transforming growth factor-beta (TGF-β)/BMP family, their receptors and regulators of signaling

**Figure 3:**
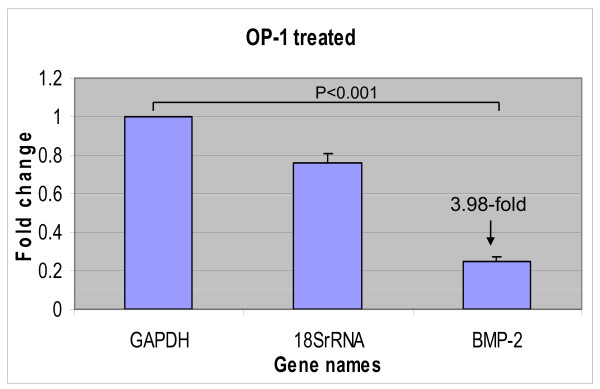
**Effect of OP-1 on BMP-2 gene expression**. Real time PCR of *in vitro *verification experiments, where knee chondrocytes cultured in monolayers were treated for 48 hours with 100 ng/ml recombinant OP-1. The graph illustrates an inhibition of BMP-2 mRNA expression.

Affimetrix analysis identified a very interesting effect of OP-1 on members of the BMP/TGF-β family (Table 7). Treatment with rhOP-1 down-regulated expression of growth differentiation factor (GDF)-15, BMP-2, and Activin A, and BMP-2 inducible kinase, while inhibition of OP-1 expression up-regulated *GDF-15 *and Activin A. Down-regulation of *BMP-2 *expression in chondrocytes treated with rhOP-1 was confirmed by real-time PCR (Figure 3). Antisense reduction of OP-1 levels resulted in down-regulation of *GDF-10 *and *TGF-α *expression (Table 7). Furthermore, a correlation was also found between OP-1 and the mediators of its downstream signaling, where OP-1AS treatment inhibited expression of transcription factors, Id proteins 2 to 4 (Table 7), binding protein Gremlin (Figure 2), and MAD genes. Changes in Id genes correlated with the earlier findings from our laboratory, which demonstrated that the treatment of chondrocytes with rhOP-1 led to the elevation of *Id1, Id2*, and *Id3 *genes and proteins [32]. Contrary to changes in the *Gremlin *gene, which showed a positive correlation with OP-1 levels, expression of Follistatin binding protein was inhibited by more than two-fold in chondrocytes treated with rhOP-1.

**Table 7:** Changes in the expression of TGF-β/BMP family related genes, their receptors, and signaling regulators

	rhOP-1 vs Cntr	rhOP-1AS vs Cntr	
	fold change	fold change	**Accession no**.
*GDF-15*	3.04↓	2.03↑	[GenBank:NM_004864]
*BMP-2*	2.67↓		[GenBank:NM_001200]
Inhibin-βa (activin A)	2.32↓	2.15↑	[GenBank:NM_002192]
BMP-2 inducible kinase	1.61↓		[GenBank:NM_017593]
Parathyroid hormone-like hormone	1.60↓	2.17↑	[GenBank:NM_002820]
*ID2*		2.32↓	[GenBank:NM_002166]
*Notch 4*		2.32↓	[GenBank:NM_004557]
*MAD-6*		2.05↓	[GenBank:NM_005585]
*Gremlin*	1.88↑	1.94↓	[GenBank:NM_013372]
*GDF-10*		1.86↓	[GenBank:NM_004962]
*ID4*	1.82↑	1.84↓	[GenBank:NM_001546]
*ID3*		1.73↓	[GenBank:NM_002167]
MAD interacting protein		1.69↓	[GenBank:NM_004799]
*MAD-4*		1.67↓	[GenBank:NM_005359]
*Notch 1*		1.65↓	[GenBank:NM_017617]
*MAD-7*		1.62↓	[GenBank:NM_005904]
*TGF-α*		1.54↓	
***Receptors***			
Frizzled homolog 10 (Drosophila)	1.57↓		[GenBank:NM_007197]
*Activin-α RI*	1.53↓		[GenBank:NM_001105]
*Activin A-RIIB*		2.42↓	[GenBank:NM_001106]
*BMPR1A*		1.83↓	[GenBank:NM_004329]
*TGF-βRI*		1.51↓	[GenBank:NM_004612]
*TGF-βRIII*		1.50↓	[GenBank:NM_003243]
*TGF-β R2*	1.58↑		[GenBank:NM_003242]
*Activin A-RIB*		1.53↑	[GenBank:NM_004302]
**Bone formation**			
Osteomodulin	1.78↑	2.56↓	[GenBank:NM_005014]

In addition, OP-1 modulated expression of the TGF-β/BMP receptors. With the exception of Activin-α RIB, which was inhibited by rhOP-1 and elevated under the lack of OP-1, expression of other receptors, Activin-α RIIB, *BMPR1A, TGF-β RI*, II, and III correlated positively with OP-1 expression (Table 7).

### Analysis of anabolic genes: other growth factors

Previously we showed that rhOP-1 stimulated expression of insulin-like growth factor (IGF)-1 and IGF-1 receptor genes [17], while inhibition of OP-1 gene expression by OP-1AS down-regulated mRNA expression of these genes. We have also documented a synergistic effect of OP-1 on IGF-1 induced responses in normal and OA chondrocytes [33,34]. Here, we confirmed an association between OP-1 and IGF-1 pathways by documenting a 1.73-fold decrease in IGF-1 receptor expression and a decrease in two IGF-1 binding proteins-5 and 7 (1.9- and 1.5-fold respectively) under OP-1AS. Furthermore, other genes within the IGF-1 signaling pathway were regulated by OP-1. Among them were *PIK3R1, PRKAR2B, MAP2K2, PDE3B*, and *SOCS3 *(Table 8).

**Table 8:** Association between OP-1 and other growth factors including igf-1, insulin, and tyrosine-kinase signaling

	rhOP-1 vs Cntr	OP-1AS vs Cntr
	fold change	fold change
*IGF-BP1*	2.17↓	
Nerve growth factor-β	1.93↓	
*VEGF-β*	1.62↓	1.50↓
Endothelial cell growth factor 1 (platelet-derived)	1.56↓	
*VEGF*	1.52↓	
Capillary morphogenesis protein 1	1.52↓	
*FGF-7*		2.87↓
*FGF-R2*	1.69↑	2.83↓
*IGF-BP5*		1.90↓
*FGF-R3*	1.87↑	1.80↓
*IGF-1R*		1.73↓
*PDGF-Rα*	1.62↑	1.70↓
*PDGF-Rβ*		1.68↓
*IGF-BP7*		1.58↓
*IRS2 *(insulin receptor substrate 2)	2.10↓	1.70↑
*DPYSL2 *(dihydropyrimidinase-like 2)	1.60↑	1.60↓
*MET *(hepatocyte growth factor receptor)	1.70↓	1.60↑
*SPRY2*: sprouty homolog 2 (Drosophila)	1.60↓	1.60↑
*SORBS1*: sorbin and SH3 domain containing 1	1.70↑	1.50↓
*PIK3R1 *(Phosphoinositide-3-kinase, regulatory subunit 1)	1.72↑	
*MAP2K2 *(mitogen-activated protein kinase kinase 2)	1.61↑	
*PDE3B *(phosphodiesterase 3B, cGMP-inhibited)	2.00↑	
*SOCS3 *(suppressor of cytokine signaling 3)	1.79↑	

Modulation of OP-1 levels affected mRNA expression of growth factors and some of their receptors that belong to various families, such as Nerve Growth Factor-β, Vascular Endothelial Growth Factor, Endothelial Cell Growth Factor 1 (platelet-derived), Capillary Morphogenesis Protein-1, and Fibroblast Growth Factor (FGF)-7. Their expression was inhibited by rhOP-1 from 1.93- to 1.5-fold. Contrary, the expression of the *FGF-R2 *and 3 receptors, and α and β receptors of Platelet-Derived Growth Factor was stimulated by rhOP-1 Table 8).

### Matrix proteins and their receptors

Cartilage-specific matrix genes were found to be modulated by rhOP-1 treatment. Expression of the collagen type IX-α3 chain and cartilage oligomeric protein (COMP) was up-regulated by about 1.5-fold in chondrocytes treated with rhOP-1 (Table 9). Among proteoglycans, versican was affected the most (by about three-fold down-regulation by OP-1AS) and syndecan was differentially regulated under both rhOP-1 and OP-1AS treatments. There were a number of other matrix genes regulated by OP-1: bone sialoprotein, osteonectin, cadherins, chondroitin sulfate PG4 and dermatan sulfate PG3 (Table 9). As expected, there was a positive correlation between OP-1 and *CD44 *expression. Inhibition of OP-1 expression resulted in 2.34-fold reduction in *CD44 *expression. However, contrary to previously published data [35], rhOP-1 inhibited hyaluronan synthase 2 expression. A number of basement membrane proteins were modulated by OP-1: α1,2,3, and five chains of collagen type IV, laminin, versican among others. Gene expression of bamacan and laminin was inhibited by rhOP-1 and stimulated under OP-1AS. OP-1 also modulated expression of collagens that are not cartilage-specific, such as collagen type I, IV, V, VI, VIII, XIV, and XVI. Their expression was inhibited under the OP-1AS treatment (Table 9). The greatest decrease in mRNA expression was found for α1 and α2 chains of type I collagen (more than 2.6-fold).

**Table 9:** Changes in the expression of matrix proteins, their receptors, and integrins

	rhOP-1 vs Cntr	OP-1AS vs Cntr	
	fold change	fold change	**Accession no**.
**Matrix proteins**			
Collagen IV-α3	1.82↓	1.54↓	[GenBank:NM_000091]
Laminin-β1	1.65↓	3.38↓	[GenBank:NM_002291]
Chondroitin sulfate PG6 (bamacan)	1.60↓	1.52↑	[GenBank:NM_005445]
Versican (chondroitin sulfate PG2)		2.98↓	[GenBank:NM_004385]
Collagen I-α1		2.63↓	[GenBank:NM_000088]
Collagen XIV-α1		2.59↓	[GenBank:NM_021110]
Collagen I-α2		2.57↓	[GenBank:NM_000089]
Cartilage associated protein		2.13↓	[GenBank:NM_006371]
Cadherin 11 (OB-cadherin (osteoblast))		2.03↓	[GenBank:NM_001797]
Collagen XVI-α1		2.00↓	[GenBank:NM_001856]
Dermatan Sulfate PG3	1.57↑	1.95↓	[GenBank:NM_004950]
Collagen V-α1		1.89↓	[GenBank:NM_000093]
Bone sialoprotein		1.83↓	[GenBank:NM_004967]
Collagen VIII-α2		1.73↓	[GenBank:NM_005202]
Collagen VI-α1		1.70↓	[GenBank:NM_001848]
Collagen IV-α1		1.70↓	[GenBank:NM_001845]
Collagen IV-α2		1.69↓	[GenBank:NM_001846]
Syndecan	1.58↑	1.67↓	[GenBank:NM_002997]
Collagen V-α2		1.65↓	[GenBank:NM_000393]
Osteonectin		1.51↓	[GenBank:NM_003118]
Cadherin 19		1.50↓	[GenBank:NM_021153]
Collagen IX-α3	1.59↑		[GenBank:NM_001853]
Cadherin	1.54↑		[GenBank:NM_001408]
COMP	1.52↑		[GenBank:NM_000095]
Collagen IV-α5		1.89↑	[GenBank:NM_000495]
Chondroitin sulfate PG4		1.64↑	[GenBank:NM_001897]
**Matrix protein receptors**			
*HAS2*	1.78↓		[GenBank:NM_005328]
*CD44*		2.34↓	[GenBank:NM_000610]
**Integrins**			
Integrin-α5	1.77↓		[GenBank:NM_002205]
Integrin-β4	1.64↓		[GenBank:AF011375]
Integrin-α6		2.34↓	[GenBank:NM_000210]
Integrin-β-like 1		2.07↓	[GenBank:NM_004791]
Integrin-β3		1.72↓	[GenBank:NM_000212]
Integrin-αE		1.70↓	[GenBank:NM_002208]

## Discussion

To the best of our knowledge, this is the first report that uses microarray analysis to provide a comprehensive understanding of the role OP-1 plays in overall cartilage homeostasis. We found that OP-1 controls cartilage homeostasis on multiple levels including regulation of genes responsible for chondrocyte cytoskeleton (*cyclin D, Talin1*, and *Cyclin M1*, for example, and confirmed in [36]), matrix production and other anabolic pathways, as well as regulation of cytokines and various catabolic pathways responsible for matrix degradation and cell death. Importantly, in many of these cases, OP-1 modulated the expression of not only the ligands, but also their receptors, mediators of downstream signaling, kinases responsible for an activation of the pathways and transcription factors that induce transcriptional responses.

Due to high variability among human samples, only a few studies have utilized microarray analysis to test the entire human genome in primary adult articular chondrocytes [4,37-39], and only one of Saas *et al. *[4] addressed in part the effect of BMP-7/OP-1. These analyses used the tissue from one or a maximum of two donor cartilage samples. In the present study, normal (grade 0) articular cartilage was collected from 12 donors within a similar age range. One of the limitations of the study is that we examined gene expression profiles only at one time point, after 48-hours of culture. Therefore, changes in early-response genes and late-response genes might have been missed. This could explain some results, as for example, the lack of changes in the expression of major cartilage matrix proteins. However, such an approach gave us a breath of the overall effects of OP-1 on cartilage homeostasis.

Due to the abundance of the results, we will discuss only the most relevant and those that could be explained by the current knowledge of the field. Perhaps most important was the finding that OP-1 is a unique growth factor in its capacity to display simultaneously pro-anabolic and anti-catabolic activities. It was previously shown that OP-1 stimulated expression and synthesis of collagen type II, aggrecan, hyaluronan, and CD44 [1,2,20,40] as well as IGF-1, IGF-1 receptor, and responses to IGF-1 [17]. In the current studies, we used high-density monolayers while in previous work explants or alginate beads were used with different media conditions (no serum vs serum or ITS-media). The finding that the microarray results shown here were comparable to the previous results suggest that the pro-anabolic effects of OP-1 in human articular chondrocytes are persistent. With regard to the anti-catabolic activity, the ability of OP-1 to counteract various pro-inflammatory/catabolic responses or directly inhibit expression of catabolic mediators was previously shown in primary chondrocyte cultures or in animal models of post-traumatic osteoarthritis or disc degeneration [17-19,24,31]. In this study, we found that OP-1 inhibits expression of IL-6 and members of the IL-6 family of chemokines as well as their receptors and signaling mediators. Furthermore, the tight association between these two classes of mediators (OP-1 and IL-6) was documented under both experimental conditions (plus or minus OP-1). Based on our new data on the role of IL-6 in acute post-traumatic responses [41], it is possible that OP-1 was able to protect cartilage from degenerative changes caused by acute trauma [31] not only due to its direct effect on matrix synthesis, but also because of its ability to inhibit IL-6, TNF-α, and the catabolic pathways induced by the fragments of the extracellular matrix: fibronectin [19], hyaluronan [20], and collagen telopeptides [42].

Another important effect of OP-1 may be an ability to inhibit expression of neuromediators and their receptors. Previously, an anti-pain effect of OP-1 was documented in the rat models of herniated disc or disc degeneration induced by injurious compression. In these studies, OP-1 injections reduced hyperalgesia and inhibited elevation of IL-1, TNF-α, substance P, bradykinin and their receptors in various disc tissues including spinal cord and dorsal ganglion [21,24,26]. In chondrocytes, it is the first report that indicates a connection between OP-1 and various neuromediators, though substance P and its NK-1 receptor were already identified in cartilage in the model of mechanical stress [43]. Very recently, our findings received another proof in phase I placebo-controlled clinical studies on OP-1 treatment for osteoarthritis patients [44], in which a single injection of OP-1 reduced pain even after six months of treatment.

Interesting results were found with regard to the ability of OP-1 to regulate the TGF-β/BMP signaling pathway. The levels of OP-1 expression positively correlated with the expression of activin-like kinase (ALK)-3 or BMP-RIA, transcription factors Id proteins 2 to 4, and a binding protein Gremlin, indicating that this could be a primary route for OP-1 signaling. We also found that another binding protein, Follistatin, exhibited a negative correlation with the levels of OP-1. Thus, our results suggest a differential regulation of these two binding proteins by OP-1, which could mean that Gremlin and Follistatin perform distinct functions in the mediating BMP responses or they are involved in different stages of signaling. This point of view is supported by studies of Tardif *et al. *[45] who reported their differential expression and spatial distribution in the dog OA model. One of the most surprising results was the finding that OP-1 inhibits expression of another member of the BMP family, BMP-2, which shares the same signaling machinery and in many cases exhibits similar anabolic activities [23,46]. This result was confirmed by real-time PCR and definitely warrants further studies in understanding the responses induced by homologous, yet very different members of the same family [16].

Finally, another unexpected result was the inhibition of TIMP-3 expression by rhOP-1; while previously, TGF-β has been shown to induce this inhibitor [47]. The differences in the results could be attributed to distinct culture conditions (primary chondrocytes in our case and passaged chondrocytes as in Qureshi *et al. *[47], assessment at various time points, or distinct signaling mechanisms between TGF-β and OP-1 that are responsible for induction of TIMP-3 expression. However, on the protein level it has already been reported that OP-1 inhibits TIMP-3 protein [48]. Furthermore, changes in TIMP-3 were parallel to changes in certain genes responsible for apoptosis, which supports the notion that in cancer cells TIMP-3 may promote cell death by apoptosis [28]. On the other hand, TIMP-3 has been shown to inhibit aggrecanase-mediated release of glycosaminoglycans in bovine nasal cartilage [49]. At this point, the role of TIMP-3 in human chondrocytes and its regulation by various mediators remains to be investigated.

## Conclusions

This analysis of gene array data strongly suggests a critical role of OP-1 in human cartilage homeostasis. OP-1 regulates numerous metabolic pathways that are not only limited to its anabolic function, but also to its anti-catabolic activity. Understanding of OP-1 function in cartilage will provide strong justification for the application of OP-1 protein as therapeutic treatment for cartilage regeneration and repair.

## Abbreviations

ADAM: a disintegrin and metalloproteinase; ALK: activin-like kinase; AP-1: activator protein-1; AS: antisense; Bcl-2: B-cell lymphoma 2; BMP: bone morphogenetic proteins; cDNA: complementary DNA; DMEM: Dulbecco's modified Eagle's medium; FBS: fetal bovine serum; FGF: fibroblast growth factor; GDF: growth differentiation factor; IGF: insulin-like growth factor; IL: interleukin; LIF: leukemia inhibitory factor; MAP kinase: mitogen activated protein kinase; MMP: matrix metalloproteinase; NCBI: National Center for Biotechnology Information; NF-κB: nuclear factor kappa-light-chain-enhancer of activated B cells; OA: osteoarthritis; ODNs: oligonucleotides; OP-1: osteogenic protein-1; PCR: polymerase chain reaction; PG: proteoglycan; PSF: penicillin/streptomycin/fungizone; rhOP-1: recombinant OP-1; RNA: ribonucleic acid; Runx1: runt-related transcription factor 1; STATs: signal transducers and activators of transcription; TGF-β: transforming growth factor-beta; TIMP: tissue inhibitor of metalloproteinases; TNF-α: tumor necrosis factor-alpha; VEGF: vascular endothelial growth factor.

## Competing interests

Stryker Biotech provided partial research support and reagents. SC served on the scientific advisory board of Stryker Biotech in 2005-2006. DCR, co-author of the manuscript, was an employee of Stryker Biotech at the time the data were collected; he has retired and no longer works at Stryker Biotech. The other authors declare that they have no competing interests.

## Authors' contributions

SC was the principle investigator on the project, developed conceptual idea, wrote the research proposal, obtained research funding and IRB approval, oversaw the progress of the study and acquisition of the project related data, coordinated efforts of the participants, wrote progress reports to the funding agencies (NIH, Stryker Biotech and Ciba-Geigy Endowed Chair), and was involved in the writing of the manuscript.

LO was the research assistant in the laboratory of Dr. Susan Chubinskaya. She performed all validation experiments, starting from cell culture and real-time PCR, analyzed gene array data and prepared the first draft of the manuscript. TA provided resources and performed gene expression analysis by Affimetrix gene array. SS was a postdoctoral fellow in the laboratory of the PI, Dr. Chubinskaya. He was solely responsible for all initial experiments: design of antisense oligonucleotides, cell isolation, culture, transfection, RNA isolation, and quality control of the RNA. JB is a collaborator on the project and assisted with validation experiments: probe design and real time PCR. He also was involved in the editing of the manuscript. DCR was a senior director of R&D at Stryker Biotech. He was involved in the conceptual development of the project, its objectives, specific aims, and experimental design. He also provided all necessary tools for the project: background knowledge on OP-1 gene, OP-1 cDNA library, recombinant OP-1, and OP-1 antibodies. He also was a main consultant on the project. RFL is a long-term collaborator of the PI, who is also collaborator on this project. All IGF-related experiments were done in Dr. Loeser's laboratory as a joint effort. He was also actively involved in data analysis and production of the manuscript as a final editor.
